# The global crisis of multidrug resistance: how to face healthcare associated infections without effective antibiotics?

**Published:** 2013-06

**Authors:** Caterina Mammina

**Affiliations:** Department of Sciences for Health Promotion and Mother-Child Care “G. D'Alessandro”, University, Palermo, Italy

Infections caused by multidrug resistant organisms (MDROs) are severely challenging the healthcare systems worldwide. Resistance to antibacterial drugs is growing in both Gram positive and Gram negative bacteria and is increasingly related to healthcare associated infections (HAIs). Undoubtedly, multiresistant Gram-negatives,especiallycarbapenem-resistant *Enterobacteriaceae* (CRE) and *Acinetobacterbaumannii*, represent the cutting edge of crisis today (1). Therapeutic options for these organisms are so extremely limited that old, previously forgotten drugs, such as polymixins, have come back touse in absence of robust data guiding dosage regimens or duration of treatment. Moreover, panresistant strains have always been reported (2).

Antibiotic resistance costs: the Alliance for the Prudent Use of Antibiotics (APUA)(http://www.tufts.edu/med/apua/) has estimated for United States of America alone a cost currently ranging between $24 billion and $38 billion a year.A report by the European Centre for Disease Control (ECDC) (http://www.ecdc.europa.eu/en/), focusing on four main types of infection - bloodstream, lower respiratory tract, skin and soft tissue and urinary tract infections -has highlighted that resistance is causing annually an economic burden of more than 900 million euros of extra in-hospitalcosts and about 600 million days of lost productivity. In this respect, poorer countries seem to be even more disadvantaged. The poorer the country, the larger is the proportion of its health-care budget being absorbed by the cost of antibacterial drugs: indeed, antibacterial drug resistance forces healthcare to turn from cheaper, but previously largely administered drugs, to more expensive alternatives (3).

Unfortunately, a synergistic combination of factors, including the evolving healthcare delivery policies and the shifting of patient demographics and underlying conditions towards higher risk profiles, is deeply changing the landscape of HAIs. All countries are involved in this global crisis with different weights attributable to a variety of concurrent factors. Just to name a few, poor healthcare resources, poor quality of antibiotics, and sometimes over-the-counter availability of antibiotics, are likely playing an important role in developing countries. In developed countries, financial crisis and cutting resources to healthcare systems along with the expanding population of old and chronically ill patients, are worsening the situation. Finally, travels, transfer of patients and the so-called “medical tourism” are bridging the boundaries between the developed and developing countries daily (4). Worldwide reports show that MDRO's infections are increasingly serious and are in exorably spilling over into the community, mainly through different alternative healthcare settings, such as long-term care facilities (LTCFs) (5). Additionally, elderly and immunocompromised persons with comorbidities, who are recurrently going back and forth between hospitals and LTCFs, contribute to the spread of MDROs and likely represent the largest and misunderstood reservoir of MDROs within the health care network (2, 5).

The second driving force of the crisis is the falling number of new antibacterial drugs approved for marketing: indeed, mainly in the field of the MDR Gram negative bacilli, the pharmaceutical industry is failing to deliver new antibacterials to replace those made ineffective by resistance. Indeed, along several decades,the pharmaceutical industry has funded and pursued research and development on antimicrobial drugs based on a return on investment in terms of future sales. However, in the last years, this business model has become unsustainable: the costs of new antibacterial development have increased under the pressure of heavier regulatory burdens, but the chance of success has parallelly declined by making investment in this field less and less attractive compared to other types of longer-lived therapeutic agents, particularly those prescribed for chronic illness (2, 3).

So, the mainstay of any control measure for reducing MDRO's infections is the implementation of basic precautions based upon understanding of how they are transmitted. A popular slogan is “back to basics: hand hygiene!” (http://blogs.cdc.gov/safehealthcare/?p=1646)- very reasonable, but not so easily applied. Transmission of MDROs is, indeed, influenced by many different and intertwined issues, including organizational and institutional factors. Growing evidence is accumulating about the effects of overcrowding, understaffing and bed occupancy rates on the risk of MDRO's infections. Indeed, several studies have described over crowding and understaffing as factors promoting decreased healthcare worker hand hygiene adherence and levels of cohorting, over burdened isolation facilities and increased movement of patients and staff between hospital wards (6, 7). All these factors, which have been synthesized with the term “organizational fatigue”, are associated with higher nurse-patient interaction and cross-infections rates (6, 7). So, the healthcare cost containment strategies aimed at obtaining greater efficiency by cutting the number of hospital beds, reducing the staff and increasing patient throughput, are likely causing negative side effects on patient safety and quality of healthcare, putting at risk the efforts to control and prevent MDRO's infections. This stress on the healthcare systems may, indeed, actually increase hospital costs due to rise in HAIs as a consequence of lapses in infection control practices by over burdened healthcare workers, as previously observed in some reported outbreaks (6–8). Moreover, wide spread diffusion of MDROs may result in ward closures and these, in turn, in delayed elective admissions and over crowding of related wards and emergency units, triggering vicious cycles where hygienic practices are unable to prevent new infections.

Successful prevention or interruption of epidemic MDROs have been achieved in both epidemic and non epidemic settings, by implementing rigorous measures at the local or, sometime, regional or national level (5). Evidence is sufficiently solid that following implementation of strict control measures, such as screening of new admissions in high-risk wards and isolation/cohorting of colonized and infected patients, incidence of MDRO's infections has stabilized or declined (5). To find smart approaches is imperative, but translating the huge quantity of white and gray literature into practice needs knowledge of local epidemiology which can be substantially different from place to place. According to the World Health Organization (WHO), surveillance system deficiency amplifies antibacterial drug resistance (http://www.who.int/drugresistance/about/en/index.html). International collaborative efforts would be required to enable accurate and continuous monitoring of the global spread of antibiotic-resistant bacteria. However, in developed countries, many studies have been carried out, but through limited times and in single facilities or small, poorly representative samples of hospitals. Even worse, reliable data from vast areas of Africa and Asia are scarce or absent. Effective surveillance systems aimed to obtain basic information to customize prevention and control interventions are essential and are not be considered an extra-cost. Similarly, the role and skill of the microbiology laboratories in detecting and typing MDROs are critical for infection prevention and control (5). Moreover, proponents of changes to healthcare systems towards a higher efficiency should learn to give more attention to adverse events, such as spread of MDRO's infections and consequent declining quality of care and associated costs.

Last, but not least, a more judicious policy of antibiotics use is urgently needed to be adopted on a worldwide scale. Unfortunately, there are no easy and universal answers to the question of how to prevent excessive and inappropriate use of antibiotics. However, all opportunities are to be promoted to learn from each other's experience on how to control antibiotic use through restriction policies, educational interventions addressing the issues of inappropriate prescription and unnecessary self-medication, and improving technologies to accurately and timely diagnose bacterial infections. Restriction of over-the-counter dispensing of antimicrobials without a prescription and regulation of the manufacture of drugs to assure their purity and potency are also urgent in all countries. Mean while, antibiotic over-use around the world in livestock and fish farming is not to be forgotten.

International efforts are also required to encourage pharmaceutical companies to develop new antibiotics. Recent closer attention to the problem of antimicrobial resistance from the WHO and many international and national health agencies suggests that urgency of the current situation is now better recognized. Some global initiatives have also been launched. For instance, the British Society for Antimicrobial Chemotherapy (BSAC) has recently established “Antibiotic Action” (http://antibiotic-action.com/), which is calling all interested parties – governments, healthcare professionals, industry and charities – to identify and implement solutions to “regenerate the discovery and development of antibacterial drugs”.

The time for action is today according to theWHO, to warrant cure for tomorrow.
